# History, mystery and chemistry of eroticism: Emphasis on sexual health and dysfunction

**DOI:** 10.4103/0019-5545.49457

**Published:** 2009

**Authors:** M. R. Asha, G. Hithamani, R. Rashmi, K. H. Basavaraj, K. S. Jagannath Rao, T. S. Sathyanarayana Rao

**Affiliations:** Reiki Practitioner and Freelance writer, 788/160, 18^th^ Cross, Ramanuja Road, Mysore, India; 1 Department of Dermatology, JSS Medical College, Mysore, India; 2 Department of Biochemistry and Nutrition, Central Food and Technological Research Institute, Mysore - 570 020, India; 3 Department of Psychiatry, JSS Medical College, Mysore - 570 004, India

## INTRODUCTION

The concept of ‘Mind’ as the functional element of ‘Self’ (Soul) has found a mention in the ‘Vedas’, the earliest known scriptures of human civilization. In Rigveda, Yajurveda and Atharvana veda, various aspects of the mind, emotions and mental illness and prevention of mental disorders have been elegantly described. Upanishads give an account of various states of mind, theories of perception, thought and memory. ‘The Bhagavadgita’ is well known as the essence of wisdom” contained in Vedas and Upanishads and Lord Krishna, the first psychotherapist, who modified and simplified the knowledge in a form easily understandable by common man.

Vatsyayana's classic work “Kamasutra” (Aphorisms of love) includes the three pillars of the Hindu religion “*Dharma*”, “*Artha*” and “*Kama*” representing religious duty, worldly welfare and sensual aspects of life respectively. The main theme here appears to be the expression of Indian attitude toward sex as a central and natural component of Indian psyche and life.[1]

Kalyanamalla, (the author of “Ananga Ranga”, a famous Sanskrit work) is said to have said that “all of you who read this book shall know how delicious an instrument is woman, who is capable of giving the divinist pleasures”.

In “The Tao of Love and Sex: The ancient Chinese way to ecstasy”, specific concepts in the Taoist approach to eroticism have been described in detail. A strong emphasis on female satisfaction is a key feature of the Tao of loving, achieved only through a proper balance and harmony of the forces of Yin (female) and Yang (male), the opposites in the cosmic scheme of the universe. As expounded by Chang, the Tao of loving is an explanation of the advice extended by various authorities in classical works on Taoism, that considered love making as part of the natural order of life.[2]

“The Perfumed Garden”, by Sheikh Nafzawi,[3] is the best known example of a classic Islamic sex manual. In this 16^th^ century guide, what people of that time thought were the most satisfactory characteristics of lovers and love making, have been poetically and colorfully described.

Lajjā snēhaha swaramadhurathā buddhayā yauvvanashreehi Kanthāsangaha swajanasamathā dhukhahānirvilāsaha I Dharma shāsthram suragurumathihi shouchmāchārachinthā Poornē sarvē jatharapitharē prāninām sambhavanthi II

Panchathanthra

Shyness, friendship, melodious voice, intellect, brilliance of youth, *enjoying the sensuality of women*, equanimity within the species, absence of sorrow/misery, carnal pleasure, religion, scriptures, intelligence of Brihaspathi (the teacher of Gods/Devathas), hygiene, concern about good behavior – *all these occur only when the creatures' stomach is full*.

This elegant *Subhashitha* from Vishnusharma's Panchathanthra clearly indicates the necessity of appropriate and adequate food/nutrition, a requisite for having the right mindset and power for optimum sexual performance. In modern times, Abraham Maslow's model of “Hierarchy of Needs” draws a parallel, emphasizing the key role played by nutrition in human sexuality.

Ayurveda is an ancient system of health care, originating in India, providing precise guidelines to protect “life”. This includes healthy living along with preventive and therapeutic measures related to physical, mental, social and spiritual harmony. In Aurveda, there's a systematic description of medicine or therapy called “*Vajikarana*” or “Virility Therapy” by which the man becomes capable of copulating with the woman. It also aids in nourishing the body of the person. According to Charaka[4] (author of “Charaka Samhitha”, an ancient ayurvedic text on Internal Medicine), one of the principal contributors to the art and science of Ayurveda, these medicines are said to give one the strength and potency of a horse by increasing the quantity and quality of semen, sperm count and sperm motility. The person who takes these aphrodisiacs is claimed to get erection for a longer duration. Charaka contends that the possible causes of decreased libido are (a) impotency by birth, (b) *Veeryaavarodha* - obstruction of semen as a consequence of controlling sexual urges for longer duration (c) *Shukra kshaya* - decrease in quantity of semen due to overindulgence in sexual activities. This may eventually lead to *Clibya* (impotency) if *Vajikara dravyas* (Libido enhancers) are not consumed by such persons regularly and (d) consumption of spicy, salty and hot food that increase *pitta* and destroys *Shukra* (semen).[4] Vajikarana includes oleation, purification, decoction enema and lubricating enema. Foods suggested which facilitate this therapy are milk, meat soup and boiled rice along with ghee, oil, meat juice, sugar and honey. Emphasis is placed on relaxed, cheerful, contented mindset.[4]

In an interesting study on the dietary pattern of field crickets *Teleogryllus commodus*, Maklakov *et al*,[5] reported that longevity in both female and male were maximized on a low-protein and high-carbohydrate diet, while their lifetime reproductive performances were maximized in a different way in their nutrient intake pattern. It was also observed that these creatures displayed sex-specific dietary preferences that boosted their reproductive performance. The authors concluded that sexes face constraints in their ability to reach their sex-specific dietary optima by the shared biology of food choice. This sexual dilemma over diet optimization may be a vital factor that connects the intricacies of nutrition and reproduction to aging and longevity.

The origin of eroticism can be traced to different psychosexual developmental stages, as propounded by Sigmund Freud, the founder of Psychoanalytical theory. In the oral stage, states of tension lead to a seeking for oral gratification, typified by quiescence at the end of nursing. This consists of the wish to be satiated and to reach that state of relaxation occurring at the end of sucking just before sleep sets in. Libidinal needs are thought to be predominant in the early parts of oral phase while they are mingled with more aggressive components later on, marked by intensification of aggressive drives combined with libidinal components in sadistic impulses. In the anal stage, eroticism refers to the sexual pleasure in anal functioning. In this phase, object cathexis of all libidinal energies is primarily autoerotic. The next in the sequence, the phallic stage is characterized by a primary focus of interest, stimulation and excitement in the genital area. This is associated with an increase in genital masturbation accompanied by predominantly unconscious fantasies of sexual involvement with parent of the opposite sex. The last one, genital stage, leads to an intensification of drives, particularly libidinal ones, wherein the psychological maturation of genital (sexual) functioning systems and accompanying normal systems occurs. According to Freud, there are two instincts in “id” (the unconscious part of human psyche) – “*Eros*” and “*Thanatos*” or “Life” and “Death” instincts. Eros, libido or sexual energy is “Life integrating force”. When Freud refers to “Sex”, it does not necessarily denote somatic, sensual pleasures, but love, affection, friendship and other related non-sexual expressions.

In a thought provoking study on children in foster homes, Yates[6] reports that not all children involved in incest are necessarily passive, unwilling victims. On the contrary, they may find such experiences and relationships gratifying. With continued intense genital and extragenital stimulation, in all probability, they become highly erotic. This over mature responsiveness can be considered as learned behavior, which is self-reinforcing and hard to alter. Lack of experience and unavailability of role models are ascribed to inability of the children to differentiate between affectionate touch and sensual touch. The realm of erotic arts encompasses expressions of sexual themes specifically related to the emotions and/or justified on aesthetic grounds. There have been countless instances of the artistic manifestations of eroticism in sculpture, painting, photography, drawing, film, literature and performing arts.

The (in) famous concept of “Femme Fatale” depicts woman as the one who lures men into danger, destruction and even death by means of exceedingly empting seductive charm. This image evolved in the 19^th^ century to become a stereotype in the 20^th^ century. Although this concept had been created “by men who, in all probability, felt threatened by the escape of women from male dominance to much longed for freedom”, these images appear to be representative of “Female liberation from male domination” and as “Rejection of maternity” and “Female control of her own sexuality and body”.

Today's man is under pressure to change. He is told to get in touch with his feminine (softer) side, encouraged to display his more caring nature. A woman or man is not born a woman or man, but is made into one by socio-cultural conditioning. Other factors playing a crucial role shaping human sexuality are neuroendocrinal, dietary and psychosocial in nature.[7]

Men, on average, are estimated to have 400% more erotic fantasies than women. Sex is one area where women and men are attracted to each other most intimately but are driven by motives that are worlds apart. She longs for emotional warmth and is aroused slowly while he craves passionate novelty and is aroused pretty fast.[7]

Harmones have been proved to be powerful engineers of brain and behavior. It has been observed that male have higher arousal threshold and women and men react very differently to stimuli. The male brain needs more extreme signals, stronger and intense stimulus and it craves the stimulus of adventure.[8] Consequently, males are more likely to indulge in dangerous risk-taking and impulsive action. High testosterone levels are associated with impatience, irritability and low ‘Frustration Tolerance’.[9]

The sexual drive in both women and men is influenced by testosterone, the hormone which men have 1000% more than women.[10] This implies that, the higher the testosterone levels in an individual, the more intense his sexual urge. During menstrual cycle in women, the testosterone levels peak just as the egg is produced. If her testosterone levels are high during this peak, her motivation for sex is also high throughout the cycle.

Vasopressin and oxytocin also play an important role in the social and sexual behavior of higher mammals. Oxytocin, when found in greater levels in females, affects sexual responsiveness, socialization and maternal nurturing while vasopressin in larger quantities in the males mediates sexual perseverance.[11] Vasopressin and oxytocin display differently effects in men and women. During foreplay, the male secretes vasopressin, which gives the impression that it plays a key role in the male's sexual craving. On the contrary, if this same substance is increased through artificial means in the female, her sexual receptivity drops down drastically.

Oxytocin mainly fosters nurturing and caring behavior in the female while it facilitates erectile capacity in the male, providing ecstasy and is released in larger amounts during orgasm. This burst of oxytocin in the male is said to have been responsible for the sexual after-effect of lingering sense of drowsy well-being, the glow of pleasant contentment. Oxytocin is also claimed to promote the male's nurturing behavior and his feeling affectionate towards his woman.[11]

Cholesterol is the precursor for steroid hormones like the testosterone needed for growth, and the estrogen for being feminine in women. Cholesterol is essential for the proper and smooth functioning of all the cells in the body. In normal, healthy humans, the “rate-limiting” step in the biosynthesis of testosterone is the conversion of cholesterol into a hormone called pregnenolone. This is next converted to either dehydroepiandrosterone (DHEA) or progesterone before being further degraded in a stepwise fashion to testosterone. It is being more and more recognized these days that apart from its influence on the health of heart, cholesterol has a considerable impact on the brain. A study conducted by Dr. Anita wells[12] at the center for Human Nutrition at the University of Sheffield, revealed that low fat diets lead to increased hostility and anger and made people feel depressed. The relationship between serum cholesterol has been investigated by Wei *et al*,[13] who implied that a high level of total cholesterol and a low level of high density lipoprotein cholesterol are important risk factors for ED. These findings were corroborated by the results of studies indicating the role of elevated serum lipids, especially cholesterol, in the development of ED.[14] Hyperlipidemia has been identified as an important factor in ED; HDL and total cholesterol/HDL ratio are implied as good predictors of ED.[15]

According to a study from Italy,[16] compared to men with normal blood pressure, those with high blood pressure have low levels of testosterone, and indulge in sex much less often. Having high cholesterol, pre-diabetes or high blood pressure leads to hardening of the arteries, which in turn decreases blood flow to the testicles, resulting in damaging the testicles and lowering testosterone. High blood pressure and high cholesterol levels lower testosterone, which act as predisposing factors for men with low testosterone. Such men are at increased risk for heart attacks. This implies that every impotent man should monitor his blood levels of cholesterol and diabetes, the two leading causes of heart attacks as well as impotence.

Neurotransmitters play a pivotal role in the link between risk and pleasure. Serotonin governs the neural circuits in the brain, which convert the limbic system (controlling sexual attraction, among other things) with the frontal cortex (that controls our ability to judge before taking risk). Greater concentrations of serotonin in the brain imply that a person's actions will probably be thoughtful and sensible, while lower concentrations indicate the contrary effect.[17–20] The harmonious biochemical cascade of neurotransmitters in the brain results in various sensations of pleasure.

Difference in the levels of serotonin in men and women explains, in part, the varying extents of aggressiveness in them. The exposure to xenobiotics is suggested to be a reason for the increase in the frequency of reproductive abnormalities. Xenoestrogens from the environment such as pesticides and pollutants (detergents, paints, organic solvents, phthalate metabolites etc.).; and from the dietary source including phytoestrogens such as isoflavones (especially genistein) found mainly in leguminous plants, most impotantly, in soyabean are known to exert adverse effects on the male and female reproductive systems.[21]

The activity of dopamine is vital in the hypothalamus where ‘Pleasure centres’ are located. When these are stimulated by an electric current, they produce dopamine, which in turn intensifies the effect of endorphins. Endorphins are natural opiates. Hence a surge of dopamine boosts the endorphins which give a feeling of elation.[22] This forms the basis of sexual ecstasy. It has been known that higher levels of dopamine in the brain results in increased sexual interest in males (and his performing ability) whereas serotonin displays the opposite effect.

Prof. Zuckerman[23] observed that the lack of difference between men and women of experience seeking suggests that while men are high on the more active forms of sensation seeking, women are just as open to novel experiences through the senses and lifestyle as men. This point could shed some light on why some male partners with sexual dysfunction leave their female counterparts frustrated and dissatisfied.

The important sensory signals that play a key role in erotic delights and sensual pleasures include (obviously) the sight and touch (vision and tactile senses) but equally vital is the sense of olfaction. Welcome to the tantalizing world of pheromones, the substances animals secrete which serve as invisible sexual signals to other members of the same species. Human pheromones are the most effective and natural sexual attractants. One such component in the underarm sweat of men, Androstenol, acts as a primitive form of communication which invariably turns the women on.[24] An intensely biological ace card women can use is their natural ovulation. The fatty acids in the vaginal fluids cause the special effect of heightened testosterone levels in the males around an ovulating female.

The preceding paragraphs serve as useful indicators for optimal sexual functioning. When any of these go haywire, the most probable outcome is ‘Sexual dysfunction’.

‘Sexual dysfunction’ may be defined as the impairment either in the desire for sexual gratification or in the ability to achieve it. Irrespective of which partner is dysfunctional, the enjoyment of sex by both man and woman in a relationship is adversely affected. In some cases, it may be caused by dysfunctional psychosexual adjustment and learning while in others, the cause may be organic.

According to a survey conducted by Laumann *et al*.[25] The most common complaints in case of women are lack of sexual interest, and inability to experience orgasm. In case of men, the most frequently reported complaints were premature climaxing, performance anxiety and lack of sexual interest.

Rosen and Leiblum[26] reported that depression may contribute to some cases of sexual desire disorders. It has been known that sexual arousal in both men and women depend on levels of testosterone.[27,28]

## MECHANISM OF ERECTION

**Figure 1:**
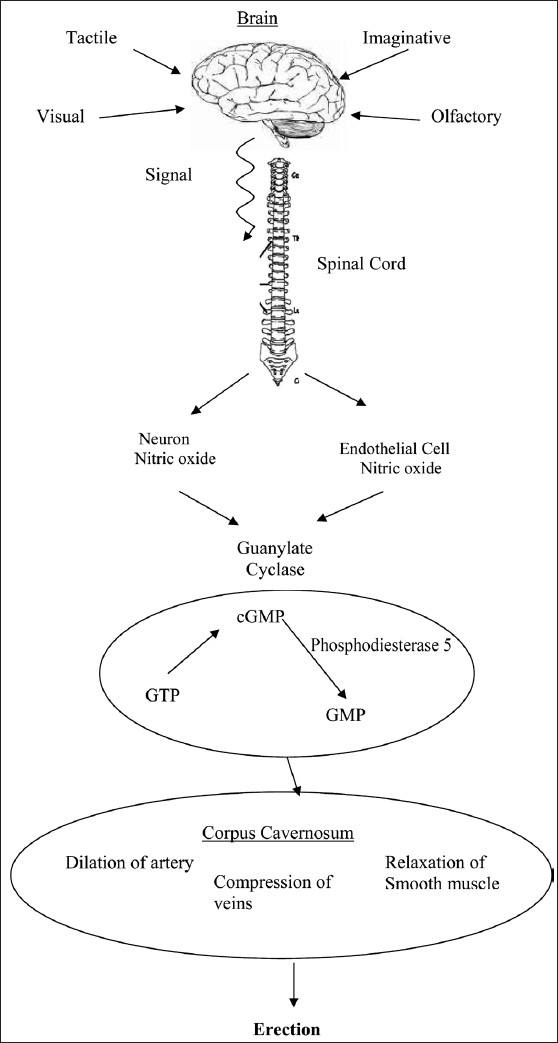
Mechanism of erection

The mechanism of erection has been covered in Figure 1, Table 1.

**Table 1:** Factors influencing sexual performance

Stressor category	Remedial approach	Therapeutic/Preventive measure
Biological	Correction of the underlying problem	Surgical intervention
Anatomical	Readjusting the delicate balance	Pharmacological
Biochemical		Physiotherapy
Hormone		
Neurotransmitter		
Physiological		
Nutritional	Correction of nutritional deficits	Supplementation of necessary nutrients. Achieving balanced diet
Psychosexual	Proper communication, improved relationship with partner on emotional level also, correction of cognitive distortions, alleviation of depression, anxiety	Sex therapy
		Psychopharmacological intervention when needed, Counseling, relaxation techniques
Psychosocial	Proper communication, social support	Family counseling, community interventions
Sociocultural	Correction of distorted attitudes in the coexisting cultural context	Counseling
Other lifestyle factors	Promoting health and sense of general well being, fostering fitness, development of habits/activities to boost confidence and self esteem	Regular exercising, cutting down/achieving total abstinence from smoking and alcohol, practice of yoga, meditation achieving relaxation
Exercise		
Smoking		
Alcohol		

Nitric oxide (NO) is known to be the principal mediator of erectile function.[29] Penile erection results from accumulation of cyclic guanosine monophosphate (cGMP) induced by NO. Gragasin *et al*,[30] proposed a similar hypothesis in rat clitorises, concluding that clitoral relaxation is caused by neurovascular-derived NO, via a protein-kinase-dependent activation of calcium-activated potassium channels. This channel appears to be an attractive target for pharmacotherapy of female ED.

Erection is a vascular phenomenon resulting from relaxation of (smooth muscle), dilation of arteries and constriction of veins.[31]

The cascade of neurological events begins with Sexual fantasy and sensory sexual stimulation resulting in impulses transmitted from the brain to the spinal cord. This leads to excitation of the central erection-related nerves of the spinal cord, which in turn transmit the commands to the corpora cavernosa. Further down the chain, the following events occur- (i) dilation of the cavernosal and helical arterioles and increase in the volume of blood in the lacunar spaces (ii) relaxation of the cavernosal smooth muscle and expansion of the vascular lacunae and finally, (iii) compression of the veins below the albuginea corpora cavernosa caused by extension of the lacunar spaces.[32]

Formerly called impotence, male erectile disorder (ED) is the inability to achieve or maintain an erection sufficient for sexual intercourse. Increased incidence of this disorder with age and erectile disorder, in general, is believed to caused by vascular disease, resulting in diminished blood flow to the organ or in decreased ability of the organ to hold blood long enough to maintain erection.[26] Smoking, obesity and alcohol abuse are some of the associated lifestyle factors. Diseases that affect the nervous system such as multiple sclerosis can also lead to erectile problems.

The female counterpart of ED, is female sexual arousal disorder, formerly known as frigidity. It is the absence of sexual arousal feelings and a marked unresponsiveness to most or all forms of erotic stimuli.

Premature ejaculation is the most prevalent male sexual dysfunction 1994.[25,33,34] Bodie *et al,*[35] reported that considerable percentage of men with ED had low testosterone, and a high percentage of patients presenting with a primary complaint of ED had increased total serum cholesterol levels.

Our body needs proper nourishment to function optimally in the three spheres – physical, mental and emotional. When the dynamic balance of brain chemicals is disturbed, various conditions such as anxiety, depression etc develop which have huge impact on sexual performance also. Neurotransmitters, the important brain chemicals, crucial for passing messages in the neural network, are made from amino acids, which we obtain through our diet. Therefore, a deficient diet can leave us unable to synthesize the chemical messengers essential for smooth and healthy functioning of the brain. On the other hand, good nutrition can supply necessary nutrients in optimal amounts, through good quality food thereby maintaining balance in the brain.

What we eat can have a direct impact on our sex life, affecting our hormones, brain chemistry, energy and stress levels. Some foods have been found to have psychoactive properties, others arouse as they are psychologically suggestive, and some can actually enhance blood flow to the genitals. These are commonly referred to as “Aphrodisiacs’. The word “aphrodisiac” has its roots in the Greek goddess of love, Aphrodite. Information on some of the common foods believed to possess aphrodisiac properties, with their associated health benefits are given below.

### Avocado

The Aztecs named the avocado tree *Ahuacuatl* meaning testicle tree. Implying the fruit hanging in pairs on the tree to male testicles. Avocados are a smooth, unctuous fruits, high in mineral content, and contain the body's master antioxidant glutathione, as well as host of healthy carotenoids, heart-friendly monounsaturated oil and superior amounts of fibre, potassium and B6.

### Chilli

They contains the chemical capsaicin that can induce the release of endorphins to create a temporary high. Capsaicin also speeds up the metabolism and increases circulation, responses that are similar to those experienced when having sex. It ignites our internal engine and stimulates our energy for passion. Eating large quantities of capsaicin may also work as an irritant to the genitals and urinary tract that can give the feelings similar to sexual excitement.

### Celery

Celery contains the male hormone androsterone that is believed to stimulate sexual arousal in women. After eating celery, androsterone is said to be released through perspiration and functions as a pheromone that turns women on. The Swedish author C.E. Hagdahl[36] states that ‘Celery contributes to a stimulation of the digestion, but is also suspected to be somewhat sexually exciting or even straightforward arousing.’ The Romans are said to have favored the abilities of this vegetable by dedicating celery to Pluto, the ‘god of sex’.

### Chocolate

Pure chocolate, the queen of natural aphrodisiacs, contains a host of compounds including “*anandamide*”, the psyochoactive “feel-good” chemical, and PEA (phenyl ethyl amine), the ‘love chemical’ which releases dopamine in the pleasure-centres of the brain and peaks during orgasm. PEA is believed to aid in inducing feelings of excitement, attraction, and euphoria. Cocoa also contains tryptophan, a key component of the neurotransmitter serotonin, known to promote a sense of relaxation and wellbeing, in addition to ‘*nature's Viagra*’ -arginine, the amino acid that enhances arousal and sensation in women and men. Arginine is converted into nitric oxide in the body which enhances blood flow and relaxes smooth muscle in the genitalia.

### Drumstick

An excellent source of minerals calcium, iron, phosphorus and magnesium, drumstick leaves and pods are used as well known folk remedy for impotence, in rural India. It is also used to strengthen eyes and brain.

### Fennel

Fennel's aphrodisiac traits have been upheld by research that has found it to increase the libido of both male and female rats. This is possibly due to the hormone-like compounds it consists of, that mimic the female hormone estrogen. This estrogenic activity is the reason why fennel has been used as a breast enlarger.

### Figs

This (sexy) fruit has long been thought of as an arousing stimulant and an open fig is believed to resemble the shape of the female sex organs. Figs have historical significance and are one of the oldest recorded fruits. A mention of figs are made in the bible (Adam and Eve wore fig leaves to cover their private parts). They are reported to be Cleopatra's favorite fruit and the ancient Greeks held them as sacred and associated them with love and fertility. A tradition followed in some Southern European countries is the throwing of figs as blessing by wedding guests at newlyweds as a sign of fertility.

### Garlic

This pungent herb is believed to be an elixir and said to stir sexual desires. Ancient Roman priestesses claimed garlic could make ‘women fall in love and men powerful’. Substantial experimental evidence has revealed that it has many disease-protective qualities such as lowering cholesterol, aiding circulation and fighting bacteria, fungi and viruses.

### Honey

Sweet, sticky honey is a good source of boron, a trace mineral that aids the body use and metabolize estrogen, the female sex hormone. Studies have suggested that this mineral may also enhance testosterone levels in the blood, the hormone responsible for promoting sex drive and orgasm in both women and men. In addition, honey contains B vitamins needed for testosterone as well as other nutrients, enzymes and phytochemicals.

### Liquorice (licorice)

This has been used in ancient China for its love and lust-provoking properties. Research has revealed that the smell itself is particularly stimulating. In a study by Dr. Hirsch, the neurological director of the Smell and Taste Treatment and Research Foundation in Chicago, black liquorice was found to enhance blood flow to the male organ by 13 per cent.[37] In China it is also reported to be particularly stimulating to women. (Smell Research Says Love Is in the Air; Smell and Taste Expert Reveals Secret Scents That Spell Attraction for This Valentine's Day Business Wire, Feb 7, 2005.

### Nutmeg

Nutmeg has been lauded as an aphrodisiac across numerous cultures and highly prized by Chinese women. It has been a common practice in India, after sumptuous meals; to chew betel leaves with coarsely powdered areca nut mixed with powdered nutmeg, grated copra, sugar etc. Research supports this use as it has been observed to increase mating behavior in mice. Large quantities of nutmeg can be psychoactive and produce hallucinogenic effects.

Herbs that are also natural aphrodisiacs, some of which are quite powerful, can have side effects and require appropriate dosage instructions.

## MALNUTRITION AND SEXUAL DYSFUNCTION

Malnutrition, associated with most diseases and disorders, certainly has an important role in sexual dysfunction.

In humans, the major impact of zinc deficiency includes changes in growth, sexual maturation and function, appetite and olfactory and gustatory sensitivities, wound healing.

In a study on impotent men under treatment for renal failure by hemodialysis, it was found that when zinc chloride was added to the dialysis solution in amounts adequate to maintain the plasma zinc levels between 100 and 150 mg/dl, significant improvement occurred in the potency of 50% of the patients. This was accompanied by an increase in testosterone and follicle stimulating hormone levels in plasma to normal range. This strongly suggests that zinc deficiency may be a reversible cause of sexual dysfunction in renal failure patients.[39] Results of another study on male subjects with oligospermia indicated that zinc supplementation resulted in higher levels of plasma testosterone and enhanced sperm count.[40]

In a study on prevalence of cardiovascular risk factors in ED, it was found that 74% of the participants had LDL Cholesterol level of greater than 120 mg%. It was suggested that presence of impotence could be an important symptom which should alert the people concerned to further investigate assessment and immediate management of possible co-existent risk factors for coronary artery disease. The investigators concluded that intervention could restore sexual function and ultimately improve cardiovascular health.[41] When remedial measures were taken to correct the elevated cholesterol levels, improvement was observed in men with organic ED.[42]

Gao *et al*,[43] studies indicated that erectile dysfunction is common among Parkinson's disease individuals. But it is unknown whether it precedes the onset of the classic features of Parkinson's disease. Their studies showed that among men who reported their erectile function before 1986, 200 were diagnosed with Parkinson's disease during 1986-2002. Further, men with early erectile dysfunction were 3.8 times more likely to develop Parkinson's disease during the follow-up than were those with very good erectile function (relative risk = 3.8, 95% confidence interval: 2.4, 6.0; p < 0.0001). In conclusion, in this retrospective analysis in a large cohort of men, the authors observed that erectile dysfunction was associated with a higher risk of developing Parkinson's disease. This study clearly indicates erectile dysfunction is not just psychosomatic but has huge clinical implications. These studies caution that if EDF is not corrected, the future health implications in terms of economics and burden on health care sector will be high. Another major challenge on clinicians is to reduce EDF in patients with cardiovascular, cancer, trauma patients. There is misconception that patient who undergone heart surgeries should not stress by having active sexual life. This concept leads to EDF in cardiovascular patients. Recently, Balami and Robertson[44] in their review indicated that Sexual dysfunction rarely threatens physical health but can take a heavy psychological toll. Sexual dysfunction is common in Parkinson's disease, occurring as a non-motor manifestation of the illness but often compounded by secondary problems relating to physical disability, psychological factors and medication effects. But recent studies over rule this thought. Further recent study by Jacobsen showed that data on hormonal factors, growth factors, comorbid conditions and lifestyle, diet, sexual life and exercise is the major risk factor for benign prostatic hyperplasia. Now EDF is a double-edged knife. If EDF is not corrected early, it is likely risk factor for Parkinson disease, prostate cancer etc. Further clinicians have greater job to correct EDF in patients who understand some medical trauma in life time, which affect their sexual activity.[45]

## LIFE STYLE AND SEXUAL DYSFUNCTION

Healthy life style factors play a pivotal role in correction and prevention of ED in men. Diabetes, Obesity, Coronary diseases are known to affect sexual functioning in men.[46] Similar observations were made in a study on women exploring the relationship between body weight, distribution of body fat and sexual function. The results indicated that obesity affected several aspects of sexuality in women with sexual dysfunction.[47]

Stress on the body or mind can affect erectile function. Because it raises blood pressure and cholesterol, stress contributes to a greater risk of ED. When they are stressed, people tend to overeat, smoke, and abuse alcohol indulging in behaviors that also place men at higher risk for ED.

The brain is the control center in the physical process of creating an erection. If the region of the brain responsible for sending the necessary impulses to the penis receives positive or negative messages, it will respond accordingly by releasing chemicals that constrict the blood vessels in the penis and inhibit the natural process that causes an erection. Even when the cause for ED is a medical illness or physical condition, most men with ED feel psychologically vulnerable and all men feel that their masculinity is threatened.

Some women can pretend to be aroused, even when they are not. Irrespective of worry, distraction or stress, many women can often feign arousal and even orgasm, and it is likely that few men can discriminate the difference. On the other hand, it is impossible for a man to feign an erection. ED is obvious and such repeated negative experiences can consequently render the very act of sex, embarrassing and humiliating for men. Faking by women of arousal and reaching orgasm, for fear of displeasing their partners or as a weak attempt to boost the fragile male ego, may ultimately lead to build up and explosion of libidinal energy in women, often resulting in infidelity and consequent break up of families, with dire psychological repercussions. An array of psychological problems such as anxiety, fear and even depression may follow. It is apparent that stress has a huge impact on sexual health and hence on the quality of life. The feeling of relaxation is the key ameliorating factor here.

Clinical studies have shown that changing or managing a variety of lifestyle factors such as cutting down smoking, controlling diabetes, avoiding substance abuse, reducing cholesterol, losing weight, following a healthy diet pattern, regularly exercising and diminishing stress and anxiety can help decrease the chances of developing impotence. Epidemiological studies indicate that promoting healthy life style changes such as these including Mediterranean-Style diets for primary prevention of the burden of sexual dysfunction at all ages yield greater benefits.[48]

The endogenous passion inducers such as pheromones, PEA, DHEA and other “Molecules of love” set the stage for romance with their “Magic of Chemistry”.[49]

Neurobiology suggests we're neurologically wired to look for romance. Researchers have shown that when individuals are shown pictures of their loved ones, areas of the brain with a high concentration of receptors for dopamine are activated. In addition to this, instrumental evidence was provided by MRI images of the brains of these individuals which suggested that the brain pattern for romantic love overlapped patterns for sexual arousal, feelings of happiness, and cocaine-induced euphoria. This unique pattern and the overlap indicate the complexity of the emotions that make up romantic love.

Sexual dysfunction in one or both partners may drive them to infidelity. Many men feel that there is a “Sense of adventure” in infidelity. Changes in life style, super busy husbands, unflattering body image in the post-birth context, lowered self-worth and frustrated ego may cause a woman to look for satisfaction and reassurance outside the marriage. Today, working couples have little time to nourish their bodies (with good nutrition) and nurture their marriages. Other contributing factors for infidelity in this group include long working hours, physical proximity to colleagues of opposite sex and easy access to tech-tools such as mobiles and on-line dating.

The management of sexual dysfunction is very important because of its significant physical and psychological morbidity. This field was largely ignored by the medical profession for many decades as it was thought not to be associated with visible morbidity. At the same time, it also carried a stigma.

Research findings have shown that our brain physically changes in response to our experiences. Novel sensations and even newer thoughts stimulate new connections between neurons. Natural approaches to gently rebalance the disturbed brain biochemistry can facilitate optimal, smooth functioning without sacrificing essential components of our “SELVES”, on the altar of emotional well being. One of the setbacks linked to prescription drugs is that they may reduce (wild) symptoms but they also smother the fire of creativity and passion within. We are humans. We need our emotions (positive and negative) to survive and evolve. Emotions are what make us essentially humans.

To achieve optimum wellness, life style changes are equally important as the dietary strategies. In addition to these, relaxation and spiritual practices play a pivotal role in restoring the balance in brain chemistry, optimizing strengths and minimizing dysfunctions. It takes more than the biochemistry of brain, sensuality of soma and symphony of psyches to reach the pinnacle of sexual bliss – the factor “X” is, adequate nutrition, regular exercise, healthy attitude towards sex and, last but not the least, the ephemeral light of love.

## References

[1] Burton R, Arbuthnot FF (1984). Translated “The Kamasutra of Vatsyayana”.

[2] Needham J (1977). The tao of love and sex: The ancient Chinese way to ecstasy. Foreword and Postscript.

[3] Burton R (1964). Translated “The Perfumed Garden” of the Sheikh Nafzawi.

[4] Charaka author of “Sushritha Samhitha” http://www.ayurhelp.com/Ayurveda_virility.htm.

[5] Maklakov AA, Simpson SJ, Zajitschek F, Hall MD, Dessmann J, Clissold F (2008). Sex-specific fitness effects of nutrient intake on reproduction and life span. Curr Biol.

[6] Yates A (1982). Children eroticized by incest. Am J Psychiatry.

[7] Anne, Moir B (1999). ‘Why men don't iron: The fascinating and unalterable differences between men and women”.

[8] (1982-1990). Trends in Alcohol-Related Traffic Fatalities- by sex- United States.

[9] Hoyenga KT, Hoyenga KB (1993). Gender-related differences.

[10] Buss DM (1994). The evolution of desire: Strategies of human mating.

[11] Panksepp J The foundations of human and animal emotions.

[12] Wells AS, Read NW, Laugharne JD, Ahluwalia NS (1998). Alterations in mood after changing to a low-fat diet. Br J Nutri.

[13] Wei M, Macera CA, Davis DR, Hormeng CA, Naudin HR, Blair SN (1994). Total cholesterol and high density lipoprotein cholesterol as important predictors of erectile dysfunction. Am J Epidemiol.

[14] Ponholzer A, Temmlc, Rauchenwald M, Maderbacher S (2006). Vascular risk factors and erectile dysfunction in a cohort of healthy men. Int J Impot Res.

[15] Rao K, Du GH, Yang WM (2005). Correlation between abnormal serum lipid and erectile dysfunction. Zhonghna Nan Ke Xue.

[16] High cholesterol causes low testosterone http://www.drmirkin.com/men/1008.html.

[17] Brown GL, Linnoila MI (1990). CSF Serotonin metabolite (5-HIAA) studies in depression, impulsivity and violence. J Clin Psychiatry.

[18] Bourgeous M (1991). Serotonin, impulsivity and suicide. Hum Psychopharmacol.

[19] Virukkunen M, Goldman D, Linnoila M (1996). Serotonin in alcoholic violent offenders. Ciba Found Sympt.

[20] Coccaro EF (1996). Neurotransmitter correlates of impulsive aggression in humans.

[21] Slowikowska-Hilczer J (2006). Xenobiotics with estrogen or antiandrogen action-disruptors of the male reproductive system. Eur J Med.

[22] Bloom FE (1985-1988). Brain, mind and behaviour.

[23] Zuckerman M (1994). Behavioral expressions and biosocial bases of sensation seeking.

[24] Cowley JJ, Brooksbank BW (1991). Human exposure to putative pheromones and changes in aspects of social behaviour. J Steroid Biochem Mol Biol.

[25] Laumann ED, Gagnon JH, Michael RT, Michaels S (1994). The social organization of sexuality: Sexual practices in the United States.

[26] Rosen RC, Leiblum SJ (1987). Current approaches to the evaluation of sexual desire disorders. J Sex Res.

[27] Sherwin BB (1988). A comparative Analysis of the role of androgen in human male and female sexual behavior; behavioral specificity: Critical thresholds and sensitivity. Special Issue: Sexual differentiation and gender-related behaviors. Psychobiology.

[28] Alexander GM, Sherwin BB (1993). Sex steroids, sexual behavior, and selective attention for erotic stimuli in women using oral contraceptives. Psychoneuroendocrinology.

[29] Kakiailatu FA (2000). The role of nitric oxide in the mechanism of penile erection. Clin Hemorheol Microcirx.

[30] Gragasin FS, Michaelakils ED, Hogan A, Mondgil R, Hashimoto K, Wu X (2004). The neurovascular mechanism of clitoral erection" Nitric oxide and cGMP-stimulated activation of BkCa channels. FASEB J.

[31] Wespes E (2002). Smooth muscle pathology and erectile dysfunction. Int J Impot Res.

[32] Nagao K, Miura K (1997). Regulation of male sexual function. Nippon Rinsho.

[33] Segraves RT, Althof S (1998). Psychotherapy and pharmacotherapy of sexual dysfunctions.

[34] Nathan P, Gorman J A guide to treatments that work.

[35] Bodie J, Lewis J, Schow D, Monga M (2003). Laboratory evaluations of erectile dysfunction: An evidence based approach. J Urol.

[36] Hagdahl CE (1879). “Cooking as Science and Art.

[37] http//www.astro.wisc.edu/mukluk/perfume.

[38] Philip A, Walravens MD (1979). Zinc metabolism and its implications in clinical medicine: Symposium on clinical nutrition. West J Med.

[39] Antoniou LD, Shalhoub RJ, Sudhakar T, Smith JC (1977). Reversal of uraemic impotence by zinc. Lancet.

[40] Hartoma TR, Nahoul K, Netter A (1977). Zinc: Plasma androgens and male sterility. Lancet.

[41] Walxzak MK, Lokhandwala N, Hodge M, Bay AT (2000). Prevalence of cardiovascular risk factors in erectile dysfunction. J Gend Specif Med.

[42] Saltzman EA, Guay AT, Jacobson J (2004). Improvement in erectile dysfunction in men with organic dysfunction by correction. J Urol.

[43] Gao X, Chen H, Schwarzschild MA, Glasser DB, Logroscino G, Rimm EB Erectile function and risk of Parkinson's disease. Am J Epidemiol.

[44] Balami J, Robertson D (2007). Parkinson's disease and sexuality. Br J Hosp Med (Lond).

[45] Hordern A (2008). Intimacy and sexuality after cancer: A critical review of the literature. Cancer Nurs.

[46] Esposito K, Giugliano G, Di Paloc, Giugliano G, Mrfella R, D'Andra F (2004). Effect of life style changes on erectile dysfunction in obese men: A randomized controlled trial. J Am Med Assoc.

[47] Esposito K, Cilotola M, Giugliano F, Bilsogni C, Schisano B, Autorino R (2007). Association of body weight with sexual function in women. Int J Impot Res.

[48] Giugliano D, Giugliano F, Esposito K (2006). Sexual dysfunction and the Mediterranean diet. Public Health Nutr.

[49] Crenshaw TL (1996). The alchemy of love and lust.

